# Draft Genome Sequence of 11399, a Transformable Citrus-Pathogenic Strain of *Xylella fastidiosa*

**DOI:** 10.1128/genomeA.01124-16

**Published:** 2016-10-13

**Authors:** Bárbara Niza, Marcus V. Merfa, Valquíria C. Alencar, Fabiano B. Menegidio, Luiz R. Nunes, Marcos A. Machado, Marco A. Takita, Alessandra A. de Souza

**Affiliations:** a Instituto Agronômico, Centro de Citricultura Sylvio Moreira, Cordeirópolis, São Paulo, Brazil; b Universidade Estadual de Campinas, Departamento de Genética, Evolução e Bioagentes, Campinas, São Paulo, Brazil; c Núcleo Integrado de Biotecnologia, Universidade de Mogi das Cruzes, Mogi das Cruzes, São Paulo, Brazil; d Centro de Ciências Naturais e Humanas, Universidade Federal do ABC, Santo André, São Paulo, Brazil

## Abstract

The draft genome of *Xylella fastidiosa* subsp. *pauca* strain 11399, a transformable citrus-pathogenic strain, is reported here. The 11399 genome size is 2,690,704 bp and has a G+C content of 52.7%. The draft genome of 11399 reveals the absence of four type I restriction-modification system genes.

## GENOME ANNOUNCEMENT

*Xylella fastidiosa* is a Gram-negative bacterium restricted to the xylem of infected plants and to the foregut of insect vectors (1). Different isolates of *X. fastidiosa* cause diseases in a wide range of economically important crops worldwide (2). In Brazil, it is responsible for causing citrus variegated chlorosis (CVC), a disease that has significantly damaged the Brazilian citrus industry (3). Due to its importance, the CVC-associated strain 9a5c of *X. fastidiosa* subsp. *pauca* was the first plant pathogenic bacterium to have its genome entirely sequenced, providing a framework for subsequent functional genomics studies (4). However, genetic manipulation of strain 9a5c is difficult and, as a consequence, experiments aimed at characterizing pathogenicity/virulence genes by gene knockout/overexpression approaches could not be performed with this strain.

Nonetheless, another citrus-pathogenic strain of *X. fastidiosa*, strain 11399 (5), has been recently reported to be transformable, opening new possibilities for studies on the biology and host interactions of this bacterium (6–9). This finding provides the opportunity to understand the genetic mechanisms that determine *X. fastidiosa* pathogenicity during the development of CVC, using transformants derived from direct genetic manipulation of strain 11399. Thus, we hereby report the draft genome of *X. fastidiosa* subsp. *pauca* strain 11399, to be used as a reference for such experiments.

For sequencing, *X. fastidiosa* strain 11399 was grown in periwinkle wilt medium (10) for 7 days at 28°C, and total genomic DNA was extracted using a DNeasy blood and tissue kit (Qiagen, Inc., Valencia, CA, USA). The genomic library construction and whole-genome sequencing was performed by Macrogen (Seoul, Republic of Korea) in an Illumina HiSeq 2000 platform (Illumina, Inc., San Diego, CA, USA), generating 40,407,960 paired-end reads (101 bp each). The 2,690,704 bp (70× coverage; G+C content, 52.7%) draft genome of strain 11399 was assembled into 35 contigs (ranging from 479 bp to 331,284 bp) by the referenced assembly method, using the Burrows-Wheeler aligner “MEM” algorithm (BWA-MEM) version 0.7.9 (11) and *X. fastidiosa* strain 9a5c as the reference genome. In addition, a plasmid sequence (45,356 bp in 1 contig) that is most similar to the 9a5c plasmid pXF51 (4) was also found in 11399. The genome of strain 11399 is likely nearly (approx. 98.5%) complete, as the genomes of *X. fastidiosa* strains range from 2.39 to 2.73 Mbp (12). Annotation was performed through submission to the NCBI Prokaryotic Genome Automatic Annotation Pipeline (PGAAP), which identified 2,248 open reading frames and 57 RNA genes.

Comparative analysis between the genomes of strains 11399 and 9a5c were performed using Mauve version 2.4.0 (13) and revealed that strain 11399 lacks four genes of the type I restriction-modification system (two specificity determinants [XF2722 and XF2726] and two DNA methylases [XF2723 and XF2724]; http://www.lbi.ic.unicamp.br/xf), which protect bacterial cells against foreign DNAs (14). The lack of these genes could thus contribute to enhance transformation efficiency in strain 11399, when compared to strain 9a5c.

### Accession number(s).

This whole-genome shotgun project has been deposited in DDBJ/ENA/GenBank under the accession number JNBT00000000. The version described in this paper is the first version, JNBT01000000.
